# Identification of Fatty Acid, Lipid and Polyphenol Compounds from *Prunus armeniaca* L. Kernel Extracts

**DOI:** 10.3390/foods9070896

**Published:** 2020-07-08

**Authors:** Soukaina Hrichi, Francesca Rigano, Raja Chaabane-Banaoues, Yassine Oulad El Majdoub, Domenica Mangraviti, Davide Di Marco, Hamouda Babba, Paola Dugo, Luigi Mondello, Zine Mighri, Francesco Cacciola

**Affiliations:** 1 Laboratory of Physico-Chemistry of Materials, Faculty of Sciences of Monastir, University of Monastir, Monastir 5000, Tunisia; soukaina.hrichi@gmail.com (S.H.); zinemighri@yahoo.fr (Z.M.); 2 Department of Chemical, Biological, Pharmaceutical and Environmental Sciences, University of Messina, 98168 Messina, Italy; youladelmajdoub@unime.it (Y.O.E.M.); dmangraviti@unime.it (D.M.); pdugo@unime.it (P.D.); lmondello@unime.it (L.M.); 3 Laboratory of Medical and molecular Parasitology-Mycology (LP3M), Faculty of Pharmacy of Monastir, Department of Clinical Biology, University of Monastir, Monastir 5000, Tunisia; raja.chaabane@laposte.net (R.C.-B.); hamouda.babba@gnet.tn (H.B.); 4 Chromaleont s.r.l., c/o Department of Chemical, Biological, Pharmaceutical and Environmental Sciences, University of Messina, 98168 Messina, Italy; davide.dimarco@chromaleont.it; 5 Department of Sciences and Technologies for Human and Environment, University Campus Bio-Medico of Rome, 00128 Rome, Italy; 6 BeSep s.r.l., c/o Department of Chemical, Biological, Pharmaceutical and Environmental Sciences, University of Messina, 98168 Messina, Italy; 7 Department of Biomedical, Dental, Morphological and Functional Imaging Sciences, University of Messina, 98168 Messina, Italy; cacciolaf@unime.it

**Keywords:** triacylglycerols, fatty acids, polyphenolic compounds, *Prunus armenica* L., apricot kernel oil, LC–MS, GC–FID/MS

## Abstract

Apart from its essential oil, *Prunus armeniaca* L. kernel extract has received only scarce attention. The present study aimed to describe the lipid and polyphenolic composition of the dichloromethane, chloroform, ethyl acetate, and ethanol extracts on the basis of hot extraction, performing analysis by gas chromatography and high-performance liquid chromatography coupled with mass spectrometry. A total of 6 diacylglycerols (DAGs) and 18 triacylglycerols (TAGs) were detected as being present in all extracts, with the predominance of OLL (dilinoleyl-olein), OOL (dioleoyl-linolein), and OOO (triolein), with percentages ranging from 19.0–32.8%, 20.3–23.6%, and 12.1–20.1%, respectively. In further detail, the extraction with ethyl acetate (medium polarity solvent) gave the highest signal for all peaks, followed by chloroform and dichloromethane (more apolar solvent), while the extraction with ethanol (polar solvent) was the least efficient. Ethanol showed very poor signal for the most saturated TAGs, while dichloromethane showed the lowest percentages of DAGs. Accordingly, the screening of the total fatty acid composition revealed the lowest percentage of linoleic acid (C18:2n6) in the dichloromethane extract, which instead contained the highest amount (greater than 60%) of oleic acid (C18:1n9). Polyphenolic compounds with pharmacological effects (anti-tumor, anti-coagulant, and inflammatory), such as coumarin derivative and amygdalin, occurred at a higher amount in ethyl acetate and ethanol extracts.

## 1. Introduction

*Prunus armeniaca* L., known as the “apricot”, belongs to the genus *Prunus* of the sub-family Prunoideae and the family Rosaceae [1]. It is native to China, and was later introduced around the Mediterranean basin [2]. Apricot is one of the oldest known oil seed crops, and it plays an important role in the health and vitality of humans. Oil extracts from the kernel of the plant *P. armeniaca* L. have shown a remarkable pharmacological effect, including high free radical scavenging capacity (antioxidant) [3,4,5]; inhibitory activity against several enzymes in an tumor development experiment [5]; and antinociceptive [6], antimicrobial [4], anticancer [7], anti-inflammatory [8], hepato-protective [9], and cardioprotective activities [10]. The large amount health benefits of *P. armeniaca* L. kernel begs the investigation of its chemical composition, thus leading to the identification of polyphenols, lipids, carotenoids, organic acids, amygdalin, and mineral elements. In particular, different classes of polyphenols, including phenolic acids, flavonoids, and antocyanins, have been positively identified.

This seed oil, as in the case of other vegetable oils, is mainly constituted of lipids, including a high proportion of triacylglycerols (TAGs) [11]. TAGs, the most abundant lipids in nature, are triesters of fatty acids (FAs) with glycerol. In particular, each of the three positions of glycerol may be occupied by different FAs. The sum of all possible combinations of FAs makes the oil a particularly complex mixture of TAGs, which asks for advanced analytical techniques for a detailed elucidation. Until now, several studies have investigated the FA profile of *P. armeniaca* L. kernel oil [12] after a trans-esterification procedure [12,13,14,15,16,17,18,19,20], wherein only two of them reported the native TAG composition [14,15], as they are effectively assumed by humans. The most abundant identified FAs were oleic and linoleic acids, followed by palmitic and stearic acids, and then the major TAGs derived from the combination of these FAs, such as triolein, dioleyl-linolein, dioleyl-palmitin, and dilinoleyl-olein. Moreover, only few studies on the characterization of chemical composition of *P. armeniaca* L. cultivated in Tunisia were published. The fruit of Tunisian *P. armeniaca* L. has been studied as a source of carotenoid compounds [21], with the kernel flour being recommended as a protein source [22]; however, studies on the chemical composition of Tunisian *P. armeniaca* L. kernel extract have not been reported until now.

The extraction of bioactive compounds from plant material have been increasingly undertaken in the last decade in order to better understand their beneficial properties [23]. Typically, bioactive compounds, such as carotenoids, polyphenols, and lipids are extracted by using a mixture of two or three solvents: polyphenolic compounds are commonly extracted through the well-known Montedoro method, employing methanol/water [24]; carotenoids are isolated by using more apolar solvents, such as hexane, ethyl ether, ethyl acetate, and acetone [25]; while lipids are commonly obtained by the well-known Folch method [26], which recommends chloroform/methanol as an extraction mixture. Specifically, for lipids and carotenoids, the use of different polarity solvents is mandatory to maximize the recovery for both polar (phospholipids and xantophills) and apolar compounds (TAGs and carotenes).

In the present research, we used successively four different pure solvents (dichloromethane, chloroform, ethyl acetate, and ethanol) in order to obtain four extracts with different chemical composition; specifically, ethanol extract was expected to be the most concentrated in polyphenols, immediately followed by ethyl acetate extract, while the dichloromethane and chloroform extracts were expected to contain almost solely apolar compounds. High-performance liquid chromatography (HPLC) coupled with mass spectrometry (MS) was used to elucidate the chemical composition of the four extracts, allowing for both qualitative and quantitative considerations. The reversed phase (RP) separation mechanism was selected for the analysis of both polar (polyphenols) and apolar compounds (lipids and carotenoids). In particular, polyphenols were separated on RP-HPLC by using a previously developed chromatographic method [27,28] in gradient elution with acidified water and acetonitrile as mobile phases, while a new chromatographic approach was investigated for lipid separation. In fact, a RP-HPLC method, based on the use of a C30 stationary phase, commonly used for carotenoid analysis [28,29], was applied, leading to the separation and identification of only acylglycerol compounds, whereas carotenoids were definitely not detected. Focusing on lipid separation, they are normally eluted according to the increasing partition number (PN) or equivalent carbon number (ECN), related to carbon (CN) and double bond (DB) numbers according to the following relationship: PN = ECN = CN − 2DB. Taking into account the high complexity of a TAG mixture, several co-elutions normally occur in the same PN region. Within this context, the additional aim of the present work was to investigate the retention behavior of a C30 column for TAG separation. Interestingly, the more hydrophobic nature of this column, compared to a more conventional C18, positively affected the chromatographic resolution as an effect of the increased retention, especially for low-PN compounds and positional isomers, similarly to the good resolution achieved for carotene isomers in previous works [28,29].

In addition, gas chromatography (GC) coupled to both mass spectrometry (MS) and flame ionization detector (FID) was used for the qualitative and quantitative determination of FAs obtained after transesterification of intact lipids. The quali-quantitative FA profile was helpful to support the identification of TAGs, which most likely contained the most abundant FAs (at least > 0.1% of the total content).

## 2. Materials and Methods

### 2.1. Chemicals and Reagents

Reagent grade quality ethanol, chloroform, ethyl acetate, dichloromethane, methanol, *n*-heptane, sodium methoxylated, and boron trifluoride in methanol (14% *w*/*v*) were purchased from Merck Life Science (Merck KGaA, Darmstadt, Germany).

LC–MS grade methanol, acetonitrile acetic acid, water, and HPLC grade methyl *tert*-butyl ether were also acquired from Merck Life Science (Merck KGaA, Darmstadt, Germany).

Standard of gallic acid, protocatechuic acid, coumarin, chlorogenic acid, catechin, epicatechin, and ferulic acid were purchase form Merck Life Science (Merck KGaA, Darmstadt, Germany).

### 2.2. Plant Seed Materials

Apricot kernels were purchased from a local market in Kondar (latitude 35°49′34″ N, longitude 10°38′24″ E), a rural region in the Tunisian Sahel, situated about 30 km from the northwest of Sousse governorate. They were milled using an electric grinder (Moulinex AR1100, France) and sieved using sieves with pore sizes of 710 μm. The powder was stored in sealed plastic bags at 4 °C until used.

### 2.3. Oil Extraction

*Prunus armeniaca* L. kernels were subjected to subsequent reflux extractions with 300 mL of four different solvents. One hundred grams of seed powder were mixed with 300 mL of dichloromethane; after 90 min under reflux, the solid particles were filtered using a filter paper, thus obtaining the residue I and dichloromethane extract. From the filtered extract, dichloromethane was evaporated, yielding a pure oil extract. Residue I was subjected to the extraction with chloroform solvent to obtain residue II and the chloroformic extract. The latter was evaporated to obtain the pure oil, while residue II was extracted with ethyl acetate solvent to obtain residue III and the ethyl acetate extract oil. Finally, residue III was mixed with 300 mL of ethanol, and the ethanol extract was obtained and evaporated to dryness. Extracts were stored at 4 °C until used. Extractions were performed in triplicate.

### 2.4. Fatty Acid Methyl Ester (FAME) Preparation

Twenty milligrams of each kernel extract were added to 500 µL of sodium methoxylated in methanol (0.5% *w*/*v*) and mixed for 120 s by using a digital shaker (IKA-Werke GmbH and Co. KG, Staufen, Germany) at 2000 rpm. The solution was heated for 15 min at 95 °C. Then, 500 µL of boron trifluoride diluted in methanol (14% *w*/*v*) was added to the reaction mixture, which was shaken for 120 s at 2000 rpm and heated for 15 min at 95 °C. Afterwards, 350 µL of *n*-heptane and 250 µL of saturated NaCl solution were added to the mixture; after 120 s of vortex mixing and 5 min of incubation, the upper heptanic phase was injected into the GC systems.

### 2.5. Sample Preparation for TAG Analysis

Thirty milligrams of each kernel extract were dissolved in 1 mL of a methanol/methyl *tert*-butyl ether (*v*/*v*) solution. The resulting solutions were filtered through a 0.45 μm Acrodisc nylon membrane (Merck Life Science, Merck KGaA, Darmstadt, Germany), prior of the injection into the HPLC- MS system *via* atmospheric pressure chemical ionization (APCI) interface.

### 2.6. Sample Preparation for Polyphenol Analysis

Dichloromethane and chloroform extracts were dissolved in chloroform (10 mg mL^−1^), while ethyl acetate and ethanol extracts were dissolved in methanol (10 mg mL^−1^). The resulting solutions were filtered through a 0.45 μm Acrodisc nylon membrane (Merck Life Science, Merck KGaA, Darmstadt, Germany) prior to the HPLC–MS analysis for the determination of polyphenolic compounds.

### 2.7. GC–MS Analysis of FAMEs

GC–MS analyses were carried out on a GCMS-QP2010 (Shimadzu, Duisburg, Germany) equipped with a split/splitless injector and an AOC-20i autosampler. The chromatographic column was a SLB-Il60i (30 m × 0.25 mm id, 0.20 μm film thickness) column (Merck Life Science). The temperature program was as follows: 50 °C to 280 °C at 3.0 °C/min. Injector was kept at 280 °C; injection volume was 0.2 µL with a split ratio of 1:20. Helium was used as a carrier gas at 30 cm/s linear velocity and an initial inlet pressure of 31.7 KPa (50 °C). MS parameters were as follows: mass range 40–550 amu, with an event time of 0.20 s; ion source temperature 200 °C, interface temperature 220 °C; ionization mode EI (70 eV), detector voltage 0.98 kV. The GCMS solution software (Shimadzu) was used for data collection and handling. The C4–C24 FAMEs standard solution was used for linear retention indices (LRIs) calculation to support identification of analytes. Moreover, peaks assignment was carried out on the basis of a double filter, namely, the MS similarity spectra (over 80%) and a LRIs ±10 range compared to the value reported in the commercial database used (LIPIDS Mass Spectral Library (Shimadzu)).

### 2.8. GC–FID Analysis of FAMEs

GC–FID analyses were carried out on a GC-2010 (Shimadzu) equipped with a split/splitless injector (280 °C), an AOC-20i autosampler, and an FID detector. GC column, temperature program, and carrier gas were the same as previously described for GC–MS analyses, apart from the inlet initial pressure of 99.5 kPa (constant linear velocity equal to 30 cm/s). The FID temperature was set at 280 °C, and gas flows were 40 mL/min for hydrogen and make-up gas (nitrogen) and 400 mL/min for air. Data were collected by using LabSolution software (Shimadzu). A relative quantification was also carried out. Analyses were performed in triplicate.

### 2.9. HPLC–APCI/MS Analysis of Lipid

HPLC–MS analyses were carried out by using a Nexera X2 system (Shimadzu, Kyoto, Japan) coupled to an LCMS-2020 detector equipped with an APCI source. The Nexera X2 system consists of a CBM-20A controller, two LC-30AD dual-plunger parallel-flow pumps (120.0 MPa maximum pressure), a DGU-20A5R degasser, a CTO-20AC column oven, and a SIL-30AC autosampler.

Separations were carried out on a C30 column (250 mm length × 4.6 mm inner diameter, 5 µm particle size) provided by YMC Europe (Schermbeck, Germany). Mobile phases were (A) methanol/methyl *tert*-butyl ether/water (81:15:4 v:v:v) and (B) methanol/methyl *tert*-butyl ether/water (15:81:4 v:v:v) under the following gradient program: 0–20 min, 0% B, 20–110 min, 0–100% B. The flow rate was set at 800 µL/min with oven temperature of 35 °C; injection volume was 20 µL.

MS detection was performed in full scan mode and in positive polarity with the following APCI parameters: interface temperature, 350 °C; DL (desolvation line) temperature, 300 °C; heat block temperature, 300 °C; and nebulizing gas (N_2_) and drying gas (N_2_) flows were 1.5 and 5 L/min, respectively. The range of acquisition was 200–1200 *m*/*z*, with an event time of 2 s. Data were collected by using LabSolution software (Shimadzu). A semi-quantification on the basis of peak area percentages was also carried out. Analyses were performed in triplicate.

### 2.10. HPLC–PDA–ESI/MS Analysis of Polyphenols

Analyses were carried out on a Shimadzu Prominence LC-20A system (Shimadzu, Kyoto, Italy), including a CBM-20A controller, two LC-20 AD dual-plunger parallel flow pumps, and a DGU-20A3 on-line degasser. The LC system was coupled to a photodiode array (PDA) serially connected to an LC–MS 2020 mass spectrometer by an electrospray (ESI) interface (Shimadzu, Milan, Italy). HPLC separation was performed on an Ascentis Express RP C18 column (2.7 µm, 150 mm, and 4.6 mm; Merck Life Science, Merck KGaA, Darmstadt, Germany). The mobile phase consisted of water/acetic acid (99.85/0.15 *v*/*v*, solvent A) and acetonitrile (solvent B), under the following gradient elution program: 0–5 min, 5% B; 15 min, 10% B; 30 min, 20% B; 60 min, 50% B; 70 min, 100% B. LC flow rate was 1 mL min^−1^ and injection volume was 10 μL. PDA detector was applied in the range of λ = 200–400 nm, and the polyphenol chromatograms were extracted at λ = 280 nm (sampling frequency: 40 Hz, time constant: 0.08 s). MS analysis was performed in negative and positive mode in the mass range *m*/*z* 100–800 with an event time of 0.3 sec; nebulizing gas (N_2_) and drying gas (N_2_) flow rate were 1.5 L min^−1^ and 15 L min^−1^, respectively; interface temperature was 350 °C; heat block and DL (desolvation line) temperatures were 300 °C. Data were collected by using LabSolution software (Shimadzu, Kyoto, Japan).

### 2.11. Statistical Analyses

In order to evaluate variability within the different assays, we applied descriptive statistic to our outcomes, and the software IBM SPSS Statistics (version 20.0) was used to calculate the means, confidence intervals (CI 95%), and standard deviations (SD).

## 3. Results

### 3.1. Oil Extraction

Extraction yields of extracts obtained from *P. armeniaca* L. kernels using four different solvents successively by hot extraction are presented in Table 1. Values are represented as percentage of total kernel weight (wt %). The results indicated that dichloromethane and chloroform had the highest extraction yields, with averages of 8.75 wt % and 6.13 wt %, respectively, which were about twice that of the ethyl acetate (average of 3.20 wt %) and ethanol (average of 4.53 wt %) extracts. This difference in extraction yields was due to the high content of lipids, and thus dichloromethane and chloroform, as non-polar solvents, are more selective for extracting lipids. However, the extraction yield decreased during subsequent extractions, with the exception of ethanol, which achieved a higher wt % compared to the previous ethyl acetate extraction, mainly related to the polar nature of polyphenol compounds synthesized by the plant.

### 3.2. Fatty Acid Profile

Table 2 reports the list of the 15 FAs identified in the four kernel extracts of *P. armeniaca* L., along with qualitative and quantitative information. From a qualitative point of view, spectral similarity higher than 85% was obtained, except for the species Me. C16:1n5, present in all the samples in poor amounts. As for LRIs, they perfectly matched the tabulated values with a maximum difference of six units and were essential to discriminate between isomers, e.g., oleic or vaccenic acid methyl esters (Me. C18:1n9 or Me. C18:1n7).

As for quantitative analysis, we performed a relative quantification. In many cases, the FAME percentages were similar between different samples, with the exception of palmitoleic acid methyl ester (Me. C16:1n7), and oleic and vaccenic acid methyl esters, which decreased in the subsequent extractions. On the contrary, the most unsaturated linolenic acid methyl ester (Me. C18:3n3) significantly increased from 0.12 in the first extraction with dichloromethane to 5.19 in the last extraction with ethanol. Linoleic acid methyl ester (Me. C18:2n6) increased from about 29% in the dichloromethane extract to about 40% in the three subsequent extractions with more polar solvents. Finally, it is noteworthy that high signals were present in the chromatograms of all the samples, independently from the employed solvent. This aspect will be better explained in the Section 4.

### 3.3. Acylglycerol Profile

**Figure 1:**
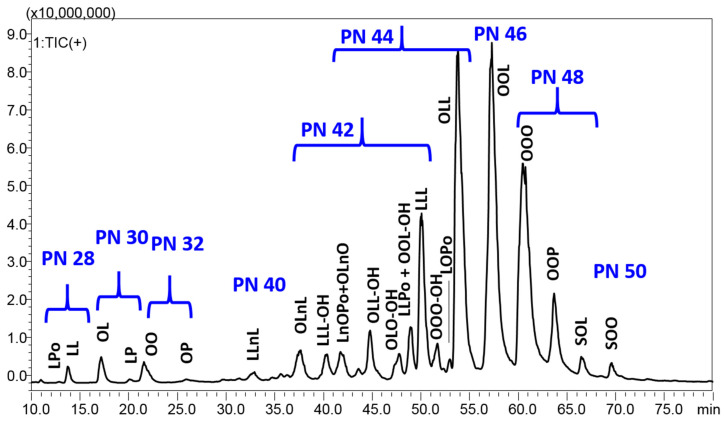
HPLC–APCI/MS analysis of the dichloromethane extract from *P. armeniaca* L. kernel. Fatty acid legend: Po, palmitoleic acid C16:1; P, palmitic acid C16:0; Ln, linolenic acid C18:3; L, linoleic acid C18:2; L-OH, hydroxylinoleic acid; O, oleic acid C18:1; O-OH, hydroxyoleic acid; S, stearic acid C18:0.

The chromatographic separation achieved for the dichloromethane extract is reported in Figure 1, which shows 70 min expansion, with a full coverage of the chromatographic space.

A total of 24 compounds with PN ranging between 28 and 50 were identified in *P. armeniaca* L. kernel extracts using the C30 colum in the RP–HPLC–APCI/MS system (Table 3). They are composed of six predominant FAs (Po, palmitoleic acid C16:1; P, palmitic acid C16:0; Ln, linolenic acid C18:3; L, linoleic acid C18:2; O, oleic acid C18:1; S, stearic acid C18:0), and are eluted according to increasing PN, starting from diacylglycerols (DAGs) in the region of PN 28–32, followed by highly unsaturated TAGs in the region of PN 40–46, characterized by a double bond number between 7 and 4, up to poorly unsaturated TAGs with PN 48–50 and only 2–3 unsaturations. Interestingly, some oxygen-containing TAGs were detected in the central region of the chromatogram. The analysis of oxidized and oxygen-containing TAGs was performed in several previous studies, paying attention to both oxidation phenomenon in fried oils through auto oxidation experiments and naturally occurring epoxides or hydroxy FAs [30,31,32,33,34,35]. All these previous works, many of which were headed by Byrdwell et al. [31,32,33,34], were used in the present research for the tentative identification of these compounds. According to their mass spectrum and retention behavior, they were tentatively identified as TAGs containing hydroxy FAs. In further detail, they were all eluted around 10 min before the corresponding non-oxidized TAGs, and were characterized by a molecular weight equal to the non-oxidized form, with 16 units added. Their fragmentation pattern was characterized by the neutral loss of water from the molecule-related ion and more DAG fragments, which are helpful for structure elucidation, particularly for the identification of the hydroxy FA. For instance, the species LLL-OH generated the fragment at *m*/*z* 599.4, corresponding to LL, and a fragment at 615.4, corresponding to LL-OH, where L-OH is hydroxylinoleic acid. On the other hand, OLL-OH produced three DAG fragments corresponding to OL (*m*/*z* 601.5), OL-OH (*m*/*z* 617.4), and LL-OH (*m*/*z* 615.4). Among such TAGs, two compounds were characterized by the same molecule-related ion, but different DAG fragments. They were tentatively identified as positional isomers, since the hydroxyl group is bound to different FAs—OLO-OH and OOL-OH were both characterized by peaks at *m*/*z* 899.7 and *m*/*z* 617.4, corresponding to the molecule-related ion and OL(OH) fragment, while the first gave the DAG fragment *m*/*z* 601.5 for OL and the second produced the fragment at *m*/*z* 603.5 for the OO DAG.

In the same way, on the basis of the relative intensity of DAG fragment ions, we were able to identify in some cases the FA placed in the stereospecific numbering sn-2 position of the glycerol backbone. To this regard, the determination of the most abundant regioisomer, arising from the combination of two or three FAs, is of great importance for the full evaluation of nutritional, biochemical, and technological properties. In fact, the lipase enzymes of living systems preferentially hydrolyzed the sn-1 and sn-3 positions of glycerol, thus generating sn-2 monoacylglycerols and free FAs, whose absorption strongly depends on their water solubility, essentially related to the length and unsaturation degree of the carbon chain. Interestingly, in the apricot oils analyzed in this work, linolenic acid (Ln, C18:3) was most probably assigned at the sn-2 position of three different TAGs, namely, LLnL, OLnL, and OLnO, in which linoleic and oleic acids were most likely bound to the external positions of the glycerol so that the ions corresponding to the DAG fragments generated by the less probable loss of the sn-2 FA were detected at very low intensities (Table 3). On the other hand, Ln occupies the external position of the LnOPo species, whose spectrum totally lacks of the ion at *m*/*z* 571 corresponding to PoLn. Palmitoleic acid (Po, C16:1) also occupies the external position in the TAG LOPo, in which oleic acid (O, C18:1) is again in the sn-2. Finally, as is already well known, saturated FAs do not occupy the sn-2 position in vegetable oils; thus, palmitic (P, C16:0) and stearic acids (S, C18:0) most probably are in the sn-1/sn-3 positions of the OOP and SOL species, as highlighted by the APCI–MS spectra. To this regard, it is worth remembering that APCI source, particularly suitable for the ionization of non-polar compounds, produces some in-source fragmentation, thus generating some diagnostic fragments [36], useful to achieve a reliable identification, without the need for MS/MS experiments, as in the present work, in which a single quadrupole MS system was employed.

All in all, six DAGs were detected between 12.87 and 25.93 min, and 19 TAGs (including the oxidized ones) were detected between 32.82 and 69.59 min. Among the 18 TAGs, LLL, OLL, OOL, OOO and OOP were the major TAGs and constituted about half of the total kernel extract TAGs. By looking at the absolute area, the extraction with ethylacetate (medium polarity) gave the highest signal for all peaks, followed by chloroform and dichloromethane, while the extraction with ethanol was the least efficient. In particular, ethanol showed a very poor signal for the highest PN TAGs, namely, OOO, OOP, SOL, and SOO, while dichloromethane showed the lowest percentage of DAGs. The oxygen-containing TAGs significantly contributed to the total composition in all the extracts. Then, their identification will need a confirmation in the near future, in order to correlate them with its potential beneficial or toxic activities.

### 3.4. Polyphenolic Profile

**Figure 2:**
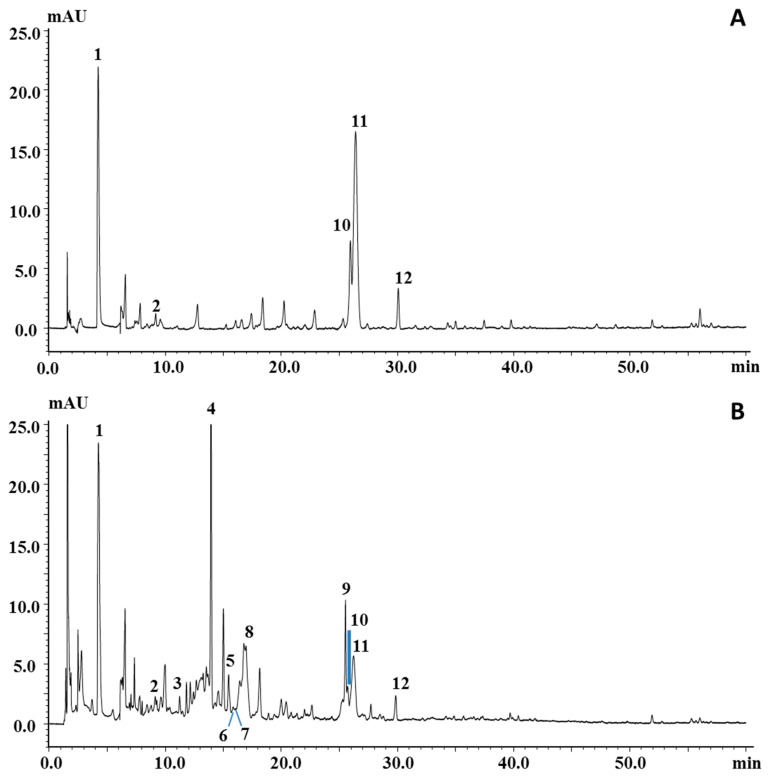
LC–photodiode array (PDA) chromatograms of polyphenolic profile of *P. armeniaca* L. kernel extracts obtained by using (**A**) ethyl acetate and (**B**) ethanol. For peak identification, see Table 4.

Figure 2 reports the PDA chromatograms of the ethyl acetate and ethanol extracts, which turned out to be the most suitable ones for polyphenol extraction; among them, the ethanol extract was the richest sample, both qualitatively and quantitatively. Peaks in the chromatograms were identified on the basis of their retention behavior, UV and MS spectra, and literature information [10,37,38,39]. In particular, 5 out of 12 peaks belong to the phenolic acid family (peaks 1–3, 5 and 10) and were all characterized by an intense ion in the negative MS spectrum corresponding to the deprotonated molecule; the larger apolar cinnamic acid derivatives, namely, chlorgenic, neochlorogenic, and ferulic acids, also showed the protonated molecules in the positive spectrum. Peak 4, the most intense peak in the chromatogram of the ethanol extract, was assigned as coumarin, due to the typical UV spectroscopic data (λ_max_ at 289 and 312 nm) and the presence of the ion at *m*/*z* 147, corresponding to the protonated molecule in the positive MS spectrum, while any signal was detected in negative mode. Peaks 6 and 7 were identified as the flavanols catechin and epicatechin, respectively, mainly due to the observation of the protonated and deprotonated molecule in the MS spectra, while their UV spectra were not quite informative. However, many of the aforementioned compounds were confirmed by standard injection, apart from neochlorogenic acid, which was indeed reported in previous works. Among the other peaks, acetylgenistin (peak 8), belonging to the isoflavone family, was tentatively identified on the basis of a parent ion at *m*/*z* 475 in positive ionization mode and the maximum UV absorption at λ_max_ 262 nm, which mostly drove the tentative identification toward this class of compounds. Peak 11, identified as amygdalin, a cyanogenic glucoside detected in negative mode as deprotonated molecule (*m*/*z* 456), has already been reported in the seed of apricot [10,39]. Peak 12 was tentatively identified as dimethoxyflavone, since it eventuated as the most probable candidate in the list generated from human metabolome database [40] for the mass *m*/*z* 281 detected in negative mode.

An unknown component was also marked in the chromatogram (peak 9) since it had quite pure MS and UV spectra. Its UV and MS spectra in both positive and negative mode are reported in Figure S1. Spectral information for all the compounds are summarized in Table 4 for each identified compound.

## 4. Discussion

Assuredly, the hot extraction method using four organic solvent of increasing polarity represented a high efficiency extraction method. A total yield of about 20% (*w*/*w*) of oil per gram of kernel was obtained, comparable or even more so than that reported in literature studies from Iran and Turkey [12,16]. The present work represents the first report on Tunisian oil extracts from *P. armeniaca* L. kernel. Extraction by solvents of different polarity allowed us to obtain different extracts containing different polarity chemical constituents. Specifically, polar solvents, such as ethanol and ethyl acetate solvent, were efficient for the extraction of lower molecular weight components, compared with the non-polar solvents, such as dichloromethane and chloroform.

As for apolar compounds, the use of a very retentive column, such as the one based on a C30 stationary phase, appeared to be the most appropriate choice to detect and satisfactorily separate both acylglycerols and eventually prenol lipids, such as carotenoids and carotenoid esters. In this specific case, we did not find prenol lipids.

The GC–FID/MS analyses of FAMEs revealed intense peaks in the chromatograms of all the samples, independently from the employed solvent. This finding is related to the wide range of lipid classes, from polar phospholipids and free FAs, soluble in polar and medium-polarity solvents, compared to apolar sterols and acylglycerols, more soluble in apolar and medium-polarity solvents, strongly depending on the unsaturation degree of the FA bound to the glycerol backbone. As a consequence, through looking at the absolute area values, rather than their percentages, the dichloromethane, ethanol, and ethyl acetate extracts were found to be quite similar, while the chloroform extract was the poorest sample. Such a finding is partially in contrast with the acylglycerol results that showed the different trend of ethyl acetate > chloroform > dichloromethane >> ethanol, which could indicate the presence of a high amount of polar lipids, such as phospholipids, which could be the object of further investigation. To this regard, a recent study regarded the determination of glycerol-phospholipids in three North African apricot (*P. armeniaca* L.) seed varieties, whose oils were extracted by the common Bligh and Dyer procedure [41]. Generally, phospholipids and glycolipids represent a minor fraction in vegetable oils commonly obtained by different extraction methods. Hence, in the current study, the analysis of the four different oils could provide unexpected results about these lipid families.

The diversity in composition of FAs is a good indicator of the stability and quality of oils. In our study, 15 different FAMEs were detected in all extracts. Conversely, a minor number of FAMEs, from 5 to 10, were identified in previous reports [10,12,16,17,18,19], which normally limit their attention to C16 and C18 FA families. The monounsaturated C20:1 FA was previously detected only by Amiran et al. [12], who performed a soxhlet extraction with hexane; on the other hand, they did not identify the ω-3 linolenic acid. The latter was reported at trace level in other works [17,18,19], with the exception of Orhan et al. [10], who reported a relative concentration around 10% of the total FA composition in some Turkish apricot oils obtained by maceration with hexane. In the present work, 5% of linolenic acid was found in the ethanol kernel extract, confirming the importance of investigating different extraction solvents to produce oils with different chemical compositions. Moreover, the C17 FAs were not reported previously; specifically, the monounsaturated C17:1 that contributed to the total composition in the same percentage than C20:1. Altogether, the dichloromethane extract was the most similar to previous reports, where oleic acid C18:1 was contained at a percentage larger than 60%, and linoleic acid C18:2 represented more than 20%, while the saturated palmitic acid was present at low content (around 5%). Such results were quite similar to those reported in previous works dealing with the determination of FAs in seed oil, obtained from apricots cultivated in Turkey [10,14,16,19,20], India [18], and Moorpark [17], thus concluding that the geographical origin has only a minimal influence on the lipid composition. Indeed, the levels of oleic and linoleic acids became more and more similar by increasing the extraction solvent polarity (both between 40% and 50% in ethyl acetate extract) up to the ethanol extract, in which, as already pinpointed, a significant amount of linolenic acid appeared. These results corroborate different literature reports about the beneficial properties of apricot seed oil [10,20]. Oleic acid, the major fatty acid identified in vegetable oils produced in the in Mediterranean countries, and at the basis of the Mediterranean diet, e.g., olive oil, presents different medicinal properties, such as reduction of inflammation and blood pressure, inhibition of cancer proliferation, and enhancement of fungicidal and bactericidal actions, while moreover exerting a prominent role in drug absorption [42]. Linoleic acid (omega-6) is an essential FA that prevents cancer and cardiovascular diseases since it is the precursor of important signaling molecules [12], and it also produces a serum cholesterol reduction [43]. However, an optimal ratio of ω-6/ω-3 FAs should always be maintained to guarantee a proper healthy status. In this regard, the role of linolenic acid as an essential ω-3 FA involved in many metabolic pathways should be taken into account [44].

The way in which all these FAs are combined in DAG and TAG structures also plays a central function in terms of oil stability [45] and biological activities, and lipolytic enzymes will have a key role to increase the FA availability in tissues [46].

In our extracts, 24 acylglycerols were identified, and OOL, OLL, OOO, and LLL were the major TAGs, in contrast to previous reports [14,15], which found OOO and OOL as the major TAGs and quantified OLL and LLL at levels less than 15% and 3%, respectively. In the present work, OLL largely overcame 20% of the total non-polar lipid composition and reached 33% in the ethanol extract; in a similar way, LLL was near 10% of the total fraction and increased to 13% in the ethanol residue. These differences could be explained by the different extraction conditions of the oil from apricot kernel. In fact, in previous works, a soxhlet extraction by petroleum ether [14] and the conventional extraction mixture chloroform/methanol [15] were employed. In fact, some differences could be related to the differences in geographical origin of the samples under investigation. Hence, future perspectives could regard the comparison between apricot kernels of different provenances or cultivars, or the application of a conventional extraction method on the same sample. It should be specified that the geographical origin could have a negligible effect on the total FA composition, while it could significantly affect the way in which FAs are combined in glycerol-phospholipids and acylglycerols, due to a different activity of specific enzymes.

As for polar compounds, 11 polyphenols consisting of 5 phenolic acids; amygdalin; coumarin; and 4 flavonoids, including 2 flavan-3-ols (catechin and epicatechin), the isoflavonoid acetylgenistin, and the flavone dimethoxylflavone, were reliably identified in this study, thanks to the combined use of PDA and MS detection. Our results showed differences compared to literature data from China [39], relative to apricot kernels of the same botanical species (*Prunus armeniaca L.*), in which amygdalin and a chlorogenic acid derivative were the major compounds, but flavanols, isoflavonoids, coumarins, and flavones were not detected. This could be related to the different extraction technologies that were based on the use of microwaves, which indeed were more efficient in the extraction of antocyanins and tannins. In another study on the characterization of kernel microwave extracts of a different *Prunus* species (*Prunus sibirica* L.), antocyanins again resulted in being the major compounds, but phenolic acids were also present at significant levels [37]. Some compounds detected at a high amount in the fruit of the same species cultivated in Croatia [47], such as catechin and epicatechin, were also detected at low levels in the seed extract of the present Tunisian cultivar, while coumarin has previously been reported in the kernel extract of a different *Prunus* species (*Prunus mahaleb* L.) cultivated in Italy [38]. All these data suggest that, as in the case of lipids, the extraction procedure plays an important role in the obtained polyphenol composition, in which, as it is already well-known, they are minor components whose biosynthesis is strongly affected by pedoclimatic factors. Hence, the comparison between different environments, extraction conditions, and apricot varieties could represent the future perspective of the present study that reported for the first time the polyphenol composition of Tunisian apricot seeds.

## 5. Conclusions

This study reports for the first time the chemical profiles (FAs, acylglycerols, and polyphenols) of the dichloromethane, chloroform, ethyl acetate, and ethanol extracts of *P. armeniaca* L. kernels cultivated in Tunisia. As expected, apricot kernels were characterized by high content of lipid compounds. Hot extraction method using four different solvents with increasing polarity was a simple and rapid method, providing useful quantitative and qualitative data. The results here reported could pave the way for the comprehensive characterization of a larger number of Tunisian apricot varieties through the determination of both polar and apolar compounds.

## Figures and Tables

**Table 1:** *Prunus armeniaca* L. kernel extract yields using four different solvents for extraction.

N	Solvent	wt % ± SD
1	Dichloromethane	8.75 ± 0.47
2	Chloroform	6.13 ± 1.78
3	Ethyl acetate	3.20 ± 0.64
4	Ethanol	4.53 ± 0.85

wt % ± SD: percentage ± standard deviation.

**Table 2:** Qualitative and quantitative determination of fatty acid methyl esters in the four extracts of *P. armeniaca* L. kernel by GC–MS and GC–FID.

Fatty Acid	Spectral Similarity *	Experimental LRI	Tabulated LRI *	Peak Area (wt % ± SD) (n = 3)			
				E13	E14	E15	E16
Me. C14:0	91%	1400	1400	0.02 ± 0.00	0.02 ± 0.00	0.02 ± 0.00	0.03 ± 0.00
Me. C16:0	92%	1603	1600	4.72 ± 0.05	5.08 ± 0.06	5.23 ± 0.04	5.37 ± 0.05
Me. C16:1n7	94%	1618	1616	0.74 ± 0.01	0.67 ± 0.01	0.66 ± 0.01	0.59 ± 0.01
Me. C16:1n5	81%	1631	-	0.01 ± 0.00	0.02 ± 0.00	0.02 ± 0.00	0.02 ± 0.00
Me. C17:0	91%	1702	1702	0.03 ± 0.00	0.03 ± 0.00	0.04 ± 0.00	0.04 ± 0.00
Me. C17:1n7	91%	1715	1718	0.11 ± 0.00	0.11 ± 0.01	0.11 ± 0.00	0.09 ± 0.00
Me. C18:0	95%	1804	1800	0.95 ± 0.03	0.98 ± 0.04	1.00 ± 0.05	1.02 ± 0.05
Me. C18:1n9	90%	1817	1810	62.38 ± 0.97	52.20 ± 1.01	49.92 ± 1.20	46.60 ± 2.07
Me. C18:1n7	93%	1823	1820	1.87 ± 0.05	1.75 ± 0.04	1.70 ± 0.03	1.69 ± 0.05
Me. C18:2n6	91%	1853	1847	28.86 ± 0.50	38.69 ± 0.85	40.48 ± 0.90	39.03 ± 1.16
Me. C18:3n3	88%	1901	1898	0.12 ± 0.00	0.17 ± 0.00	0.51 ± 0.03	5.19 ± 0.40
Me. C20:0	93%	2000	2000	0.09 ± 0.00	0.14 ± 0.00	0.15 ± 0.01	0.16 ± 0.01
Me. C20:1n9	90%	2015	2014	0.08 ± 0.00	0.09 ± 0.00	0.10 ± 0.00	0.10 ± 0.01
Me. C22:0	89%	2200	2200	0.02 ± 0.00	0.03 ± 0.00	0.03 ± 0.00	0.04 ± 0.00
Me. C24:0	88%	2400	2400	0.02 ± 0.00	0.03 ± 0.00	0.03 ± 0.00	0.04 ± 0.00

* lab-made database; legend: E13 = dichloromethane extraction, E14 = chloroform extraction, E15 = ethylacetate extraction, E16 = ethanol extraction, wt % ± SD: percentage ± standard deviation.

**Table 3:** Lipid compounds identified in the seeds of *P. armeniaca* L. extracts by LC–MS, *via* atmospheric pressure chemical ionization (APCI) interface.

R_T_ (min)	PN	Compound	Peak Area (wt % ± SD) (*n* = 3)				[M+H]^+^	[M+H-H_2_O]^+^	[M-FA+H]^+^
			E13	E14	E15	E16			
12.87	28	LPo	0.03 ± 0.00	0.06 ± 0.00	0.06 ± 0.00	0.14 ± 0.01	591.4	573.4	311.1, 337.2
13.75	28	LL	0.42 ± 0.02	1.13 ± 0.04	1.24 ± 0.05	1.51 ± 0.05	617.4	599.4	337.2
17.16	30	OL	0.95 ± 0.03	2.09 ± 0.05	1.94 ± 0.05	1.79 ± 0.03	619.4	601.4	337.2, 339.2
20.1	30	LP	0.11 ± 0.01	0.39 ± 0.03	0.23 ± 0.02	0.21 ± 0.02	575.4	-	313.1, 337.2
21.56	32	OO	1.03 ± 0.02	1.44 ± 0.04	1.10 ± 0.04	1.75 ± 0.05	603.5	-	339.2
25.93	32	OP	0.11 ± 0.00	0.46 ± 0.03	0.13 ± 0.00	0.31 ± 0.01	577.5	-	313.1, 339.2
32.82	40	LLnL	0.51 ± 0.01	1.53 ± 0.08	0.45 ± 0.05	0.18 ± 0.01	877.9	-	599.4 (very low), 597.5
37.58	42	OLnL	1.89 ± 0.09	4.36 ± 0.10	0.90 ± 0.07	0.92 ± 0.09	879.6	-	597.5, 599.5, 601.4 (very low)
40.33	42	LLL-OH	1.37 ± 0.11	1.86 ± 0.10	1.56 ± 0.10	1.48 ± 0.09	895.9	877.9	599.4, 615.4
41.73	42	LnOPo	1.72 ± 0.13	2.88 ± 0.12	0.60 ± 0.03	0.69 ± 0.04	853.7	-	575.3, 599. 4
	44	OLnO					881.6	-	599.4, 603.4 (very low)
44.69	44	OLL-OH	2.38 ± 0.15	3.96 ± 0.25	3.11 ± 0.25	2.27 ± 0.13	897.6	879.7	601.5, 615.4
47.71	46	OLO-OH	1.52 ± 0.05	1.31 ± 0.05	1.46 ± 0.05	1.14 ± 0.04	899.67	881.7	601.5, 617.4
48.98	42	LLPo	2.00 ± 0.10	2.52 ± 0.11	2.97 ± 0.13	1.92 ± 0.10	853.6		573.4, 599.5
	46	OOL-OH					899.7	881.6	603.3, 617.4
50.05	42	LLL	8.02 ± 0.09	9.96 ± 0.18	10.34 ± 0.20	13.02 ± 0.30	879.7	-	599.4
51.66	48	OOO-OH	1.76 ± 0.01	1.32 ± 0.00	2.20 ± 0.05	0.60 ± 0.00	901.6	883.6	603.5, 601.5 (-H_2_O), 619.4
52.91	44	LOPo	0.57 ± 0.00	0.66 ± 0.01	0.89 ± 0.01	0.74 ± 0.05	855.6		573.4. (very low), 575.4, 601.4
53.79	44	OLL	22.31 ± 0.92	21.68 ± 0.90	19.00 ± 0.85	32.78 ± 0.80	881.7	-	599.4, 601.4
57.27	46	OOL	23.66 ± 0.82	20.31 ± 0.79	21.15 ± 0.70	20.57 ± 0.81	883.7	-	603.5, 601.5
60.57	48	OOO	20.14 ± 0.50	13.50 ± 0.57	15.35 ± 0.48	12.10 ± 0.60	885.7	-	603.6
63.69	48	OOP	5.74 ± 0.30	4.53 ± 0.33	9.33 ± 0.32	2.72 ± 0.26	859.7	-	577.3, 603.5
66.66	48	SOL	1.57 ± 0.05	1.52 ± 0.05	3.46 ± 0.10	0.90 ± 0.08	885.8	-	601.5, 603.4 (very low), 605.5
69.59	50	SOO	1.01 ± 0.10	0.88 ± 0.05	1.47 ± 0.11	0.44 ± 0.03	887.7	-	603.5, 605.4

E13 = dichloromethane extraction, E14 = chloroform extraction, E15 = ethylacetate extraction, E16 = ethanol extraction. Fatty acid legend: Po, palmitoleic acid C16:1; P, palmitic acid C16:0; Ln, linolenic acid C18:3; L, linoleic acid C18:2; L-OH, hydroxylinoleic acid; O, oleic acid C18:1; O-OH, hydroxyoleic acid; S, stearic acid C18:0. Triacylglycerol (TAG) names are conventionally reported in order of decreasing fatty acid (FA) molecular weight when the stereospecific numbering (sn) position of FAs is unknown. wt % ± SD: percentage ± standard deviation.

**Table 4:** Polyphenolic compounds identified in the extracts from *P. armeniaca* L. kernels by LC–PDA–MS.

N.	Compound	T._R_ (min)	UV/VIS (nm)	Molecule-Related Ion	Fragments (*m*/*z*)	Reference
1	Gallic acid *	4.23	269	169 (−)	-	[37]
2	Protocatechuic acid *	9.14	259, 292	153 (−)	-	[10]
3	Neochlorogenic acid	11.24	321	353 (−), 355 (+),	310 (+)	[39]
4	Coumarin *	13.95	289, 312	147 (+)	-	[38]
5	Chlorogenic acid *	15.44	321	353 (−), 355 (+)	-	[39]
6	Catechin *	15.81	262	289 (−), 291 (+)	-	[10]
7	Epicatechin *	16.10	263	289 (−), 291 (+)	-	[10]
8	Acetylgenistin	16.74	262	475 (+)	456 (−)	-
9	Unknown	25.52	267	-	605 (−), 629 (+), 265 (+)	-
10	Ferulic acid *	25.71	290, 321	193 (−), 195 (+)	-	[10,37]
11	Amygdalin	26.22	206, 248	456 (−)	-	[10,39]
12	Dimethoxyflavone	29.85	265, 358	281	265 (+)	-

* confirmed by standard injection.

## Supplementary Materials

The following are available online at https://www.mdpi.com/2304-8158/9/7/896/s1, Figure S1: (a) UV, (b) MS negative spectrum and (c) MS positive spectrum of peak 9.
