# Supplementary figures and images for: Identification of Fatty Acid, Lipid and Polyphenol Compounds from Prunus armeniaca L. Kernel Extracts
Source: Foods. 2020 Jul 8;9(7):896. doi: 10.3390/foods9070896 (PMC7404456; doi:10.3390/foods9070896)

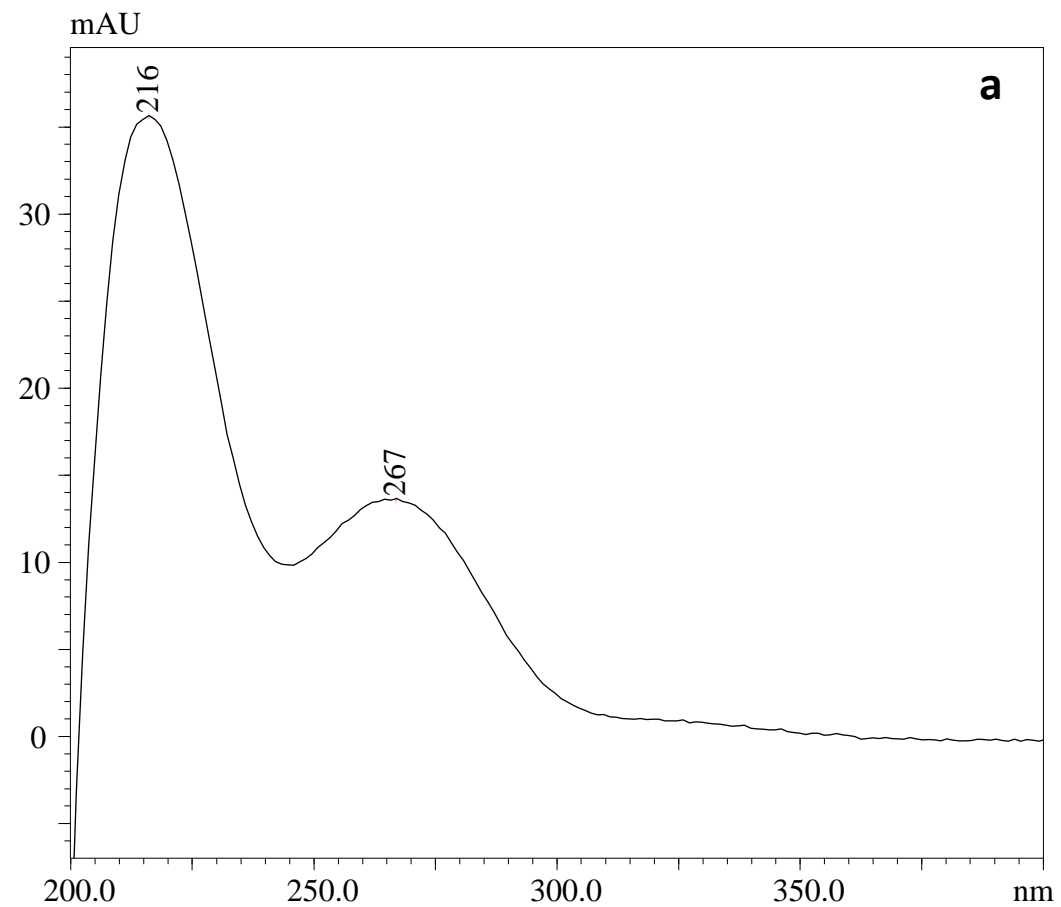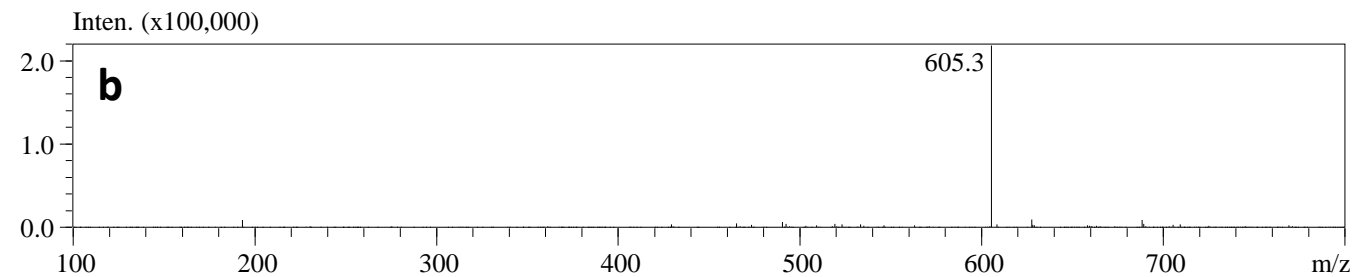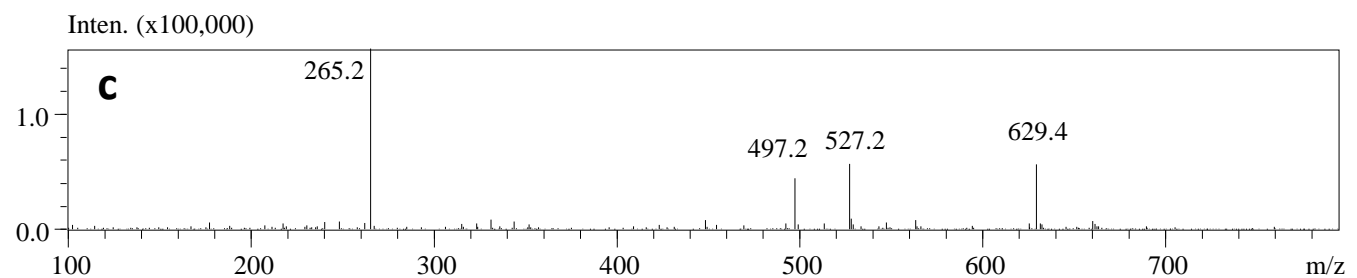

Figure S1. a) UV, b) MS negative spectrum and c) MS positive spectrum of peak 9.

Supplement: Supplementary file 1 [file foods-09-00896-s001.pdf]
